# Expansion and Functional Diversification of Long-Wavelength-Sensitive Opsin in Anabantoid Fishes

**DOI:** 10.1007/s00239-024-10181-0

**Published:** 2024-06-11

**Authors:** Jan Gerwin, Julián Torres-Dowdall, Thomas F. Brown, Axel Meyer

**Affiliations:** 1 Zoology and Evolutionary Biology, Department of Biology, University of Konstanz, Konstanz, Germany; 2 Department of Biological Sciences, University of Notre Dame, Notre Dame, IN USA; 3 Max Planck Institute of Molecular Cellular Biology and Genetics, Dresden, Germany; 4 Present Address: German Cancer Research Center (DKFZ), Division Signaling and Functional Genomics and Department of Cell and Molecular Biology, Medical Faculty Mannheim, Heidelberg University, Heidelberg, Germany; 5 Present Address: Leibniz Institute for Zoo and Wildlife Research, Berlin, Germany

**Keywords:** Gene duplication, Opsin gene, Gene expression, Siamese fighting fish

## Abstract

Gene duplication is one of the most important sources of novel genotypic diversity and the subsequent evolution of phenotypic diversity. Determining the evolutionary history and functional changes of duplicated genes is crucial for a comprehensive understanding of adaptive evolution. The evolutionary history of visual opsin genes is very dynamic, with repeated duplication events followed by sub- or neofunctionalization. While duplication of the green-sensitive opsins *rh2* is common in teleost fish, fewer cases of multiple duplication events of the red-sensitive opsin *lws* are known. In this study, we investigate the visual opsin gene repertoire of the anabantoid fishes, focusing on the five *lws* opsin genes found in the genus *Betta*. We determine the evolutionary history of the *lws* opsin gene by taking advantage of whole-genome sequences of nine anabantoid species, including the newly assembled genome of *Betta imbellis*. Our results show that at least two independent duplications of *lws* occurred in the *Betta* lineage. The analysis of amino acid sequences of the *lws* paralogs of *Betta* revealed high levels of diversification in four of the seven transmembrane regions of the *lws* protein. Amino acid substitutions at two key-tuning sites are predicted to lead to differentiation of absorption maxima (λ_max_) between the paralogs within *Betta*. Finally, eye transcriptomics of *B. splendens* at different developmental stages revealed expression shifts between paralogs for all cone opsin classes. The *lws* genes are expressed according to their relative position in the *lws* opsin cluster throughout ontogeny. We conclude that temporal collinearity of *lws* expression might have facilitated subfunctionalization of *lws* in *Betta* and teleost opsins in general.

**Supplementary Information:**

The online version contains supplementary material available at 10.1007/s00239-024-10181-0.

## Introduction

Exploring the origin of genetic diversity that eventually results in phenotypic diversity is one of the main goals of evolutionary biology. Even though the emergence of novel genetic variants is a prerequisite for selection, retention of novel genotypic diversity in protein-coding sequences is often prevented by pleiotropic effects, even if the new resulting phenotype is potentially favorable (Molodtsova et al. 2014). One way to circumvent the problem of pleiotropic effects can be the rise of new genetic material by gene duplication (Ohno 1970). In this case, the sequence and function of one paralog of the gene can be maintained, while the second copy can accumulate mutations that then are exposed to selection possibly resulting in a new function of this paralog (Rastogi and Liberles 2005).

Most gene duplications result from one of three mechanisms: retroposition, tandem duplication by unequal crossing over, or chromosomal or whole-genome duplication (Zhang 2003). The stem lineage of teleost fishes experienced a fish-specific genome duplication (Meyer and Van de Peer 2005) that resulted in the expansion of many gene families that we can still see today when comparing teleost and tetrapod genomes (Amores et al. 1998; Cortesi et al. 2021) and has been discussed to be a major factor in the success of teleost divergence (Glasauer and Neuhauss 2014; Hoegg et al. 2004). Duplication in tandem, which results in two paralogs near each other, has been a major driver of diversity in some teleost gene families, too (Lu et al. 2012; Peatman and Liu 2007; Rennison et al. 2012). However, the most common fate of the new paralog after duplication is a loss of function, as mutations that impair the gene’s original function are likely to accumulate after the release of selective pressure (Lynch and Conery 2000). Another possible fate for the duplicates is subfunctionalization (de Souza et al. 2005; Kluver et al. 2005). In this case, both copies take over part of the original function of the gene, e.g., expression during certain ontogenetic stages. This allows for the accumulation of genetic divergence among copies increasing their functional divergence and fine-tuning adaptation (Flagel and Wendel 2009; Lynch and Force 2000; Wagner 1998).

A great example of a family of genes that commonly experience duplication events with subsequent subfunctionalization is the opsin genes (Porter et al. 2012), particularly in teleost fish (Musilova et al. 2021). The visual system of teleost fishes is highly diverse, as different species have adapted to a wide range of light conditions of different aquatic environments (Carleton et al. 2020). The typical teleost genome harbors two classes of rod opsin genes. One of these genes, the extra-ocular rhodopsin (*exorh*), is not expressed in the retina and has no function in vision (Bellingham et al. 2003; Mano et al. 1999). The second class (*rh1*) is expressed in the retina and is responsible for vision in dim light. In addition, teleost fish typically retain the four vertebrate cone opsin classes responsible for color vision (*sws1*, *sws2*, *rh2*, *lws*) (Bowmaker 1998; Musilova et al. 2019; Yokoyama 2000). However, the number of genes in each of the classes can vary substantially as duplication events happened frequently during the evolutionary history of opsins (Musilova et al. 2019; Rennison et al. 2012). While a single copy of *rh1* is present in most teleost taxa, few deep-sea lineages expanded the number of *rh1* paralogs drastically in response to low levels of visible light in their environment (Musilova et al. 2019). Cone opsin genes show a more dynamic evolutionary history driven by ancient and more recent duplication events and subsequent gene losses, resulting in a plethora of different opsin repertoires across the teleost phylogeny (Bowmaker 2008; Cortesi et al. 2021, 2015; Musilova and Cortesi 2021). The UV and blue-light-sensitive genes *sws1* and *sws2* experienced the lowest degree of duplication of the cone opsins, with most species possessing one copy of *sws1* and one to three copies of *sws2* (Cortesi et al. 2015; Rennison et al. 2012). One to three copies of the green-sensitive opsin *rh2* are present in most teleost genomes (Musilova et al. 2019) with expansions to up to eight copies of the gene in some marine species (Musilova and Cortesi 2021; Musilova et al. 2019). One or two paralogs of the red-sensitive opsin *lws* can be found in most teleost genomes, but this gene has experienced recent duplication events in many different lineages resulting in four or five paralogs in the genomes of some lineages (Cortesi et al. 2021). Reconstructing the evolutionary history of cone opsin genes remains challenging due to gene conversion among paralogs (Musilova et al. 2021; Rennison et al. 2012).

The high number of paralogs within different opsin classes is putatively maintained by molecular divergence resulting in variation in spectral sensitivity of the different copies, allowing for fine-tuned changes during ontogeny or in response to environmental changes (Carleton et al. 2020; Chinen et al. 2003; Lupše et al. 2022; Torres-Dowdall et al. 2017; Ward et al. 2008). The effects of amino acid substitutions at certain positions in the opsin protein have been studied extensively allowing predictions of changes in the spectral sensitivity for some substitutions (Yokoyama 1995, 2008; Yokoyama and Jia 2020; Yokoyama et al. 2008b). The changes in spectral sensitivity after an amino acid substitution can be small (Yokoyama et al. 2008a), but the accumulation of multiple substitutions at key positions might result in major spectral sensitivity changes (Watson et al. 2011), which can help to compensate for the evolutionary losses of other opsin classes (Dulai et al. 1999; You et al. 2014). In particular, substitutions of the opsin’s residues that interact with the light-absorbing chromophore play a critical role in causing spectral shifts of the opsin (Yokoyama 2008). Amino acid substitution in opsin genes is a common mechanism by which organisms adapt to new photic conditions (Cortesi et al. 2021; Harer et al. 2018; Register et al. 1994; Rennison et al. 2012; Terai et al. 2001; Ward et al. 2008; You et al. 2014).

The possibility to change the spectrum of visual sensitivity by changing expression levels of different opsin genes also allows fast adaptation to new photic environments (Carleton et al. 2020). Even though most species only express a subset of opsins at the same time, the high diversity of opsin genes allows different teleost species to adapt their visual sensitivity to a wide range of photic environments and to changing needs throughout their lifetime. Accordingly, most teleost species express different sets of opsins throughout their ontogeny, most typically shifting from shortwave-sensitive genes during early ontogenetic stages to a set of more longwave-sensitive genes during the adult life stage (Allison et al. 2010; Carleton et al. 2008; Chang et al. 2021; Harer et al. 2017; Shand et al. 2008; Spady et al. 2006). Studies from various young lineages that inhabit new photic environments indicate that the fastest way to adapt to new photic conditions is the acquisition of fixed changes of expression of the existing opsin repertoire (Carleton et al. 2010; Carleton and Kocher 2001; Harer et al. 2018; O’Quin et al. 2010; Parry et al. 2005; Rennison et al. 2016; Torres-Dowdall et al. 2021; Torres-Dowdall et al. 2017; Wright et al. 2019). Furthermore, the presence of several opsin genes with different spectral sensitivities allows for quick plastic changes of the expressed opsin profile in response to changes in the photic environment (Harer et al. 2019, 2017; Hofmann et al. 2010; Nandamuri et al. 2017; Sakai et al. 2018). These adaptive changes in the visual system of teleosts are only made possible by the combination of frequent duplication events followed by neofunctionalization of the paralogs by amino acid substitutions.

Here, we investigate the visual system of anabantoid fishes, with a focus on the Siamese Fighting Fish, *Betta splendens*. This species was domesticated hundreds of years ago (Kwon et al. 2022) and a multitude of different color and fin morphs have been bred through artificial selection (Wang et al. 2021). *B. splendens* has been a popular model organism in behavioral biology for decades (Baenninger 1966; Bronstein 1994; Simpson 1968), but with the recent emergence of high-quality genomic information on the species, genetic studies become more abundant (Kwon et al. 2022; Wang et al. 2021; Zhang et al. 2021). In particular, we explore the evolutionary history of the visual opsin genes in Anabantiformes and examine how gene duplication has affected the evolution of amino acid sequence and expression of the different opsin genes with an emphasis on the long-wavelength-sensitive opsin (*lws*) as recent studies have shown that the genome of *B. splendens* harbors the unusually high number of five copies of that gene (Cortesi et al. 2021). Specifically, we investigated (i) the evolutionary history of opsin genes in the order Anabantiformes to determine when opsin gene duplications occurred, (ii) the role of subfunctionalization by examining sequence divergence that could result in changes in spectral sensitivity and (iii) the expression pattern of opsins in *B. splendens* during ontogeny using long-read RNA sequencing, which allows for precise mapping of reads to the highly similar paralogs of the *lws* gene.

## Methods

### Sequence Collection

Opsins were identified and extracted from genome assemblies (*N* = 4), from reads in the public archive (aligned to a reference genome) (*N* = 4), and from a new genome (*B. imbellis*). Opsin sequences were identified and retrieved from genome assemblies of eight different species in the order Anabantiformes: *Channa argus*, *Anabas testudineus*, *Helostoma temminckii*, *Betta splendens*, *Betta imbellis*, *Betta mahachaiensis*, *Betta smaragdina,* and *Betta siamorientalis*. Additionally, we retrieved the sequences of two outgroup teleosts to anchor our phylogeny: *Danio rerio* and *Amphilophus citrinellus*. Sequences of *D. rerio*, *Am. citrinellus*, *A. testudineus,* and *B. splendens* were retrieved via the search function of the ensemble genome browser (www.ensembl.org). Whole-genome sequences of *B. smaragdina* (ERR3904041), *B. siamorientalis* (ERR3904031), *B. mahachaiensis* (ERR4766262), and *H. temminckii* (ERR3332389) were available as short reads in the NCBI short-read archive (https://www.ncbi.nlm.nih.gov/sra). The short reads were aligned to the reference genome of *B. splendens* using the Burrows-Wheeler Aligner (Li and Durbin 2009) and sorted and indexed using *samtools*. Consensus sequences from the alignments were created using the *mpileup* command of *samtools*, *bcftools* and *vcfutils.pl*. Assembled reference genomes of *H. temminckii* (GCA_900302695.1) and *C. argus* (PRJNA731586) were available at the NCBI. The newly sequenced and assembled *B. imbellis* genome is described below. Sequences of species that were not available on ensemble.org were retrieved from the reference genomes using ViroBLAST (Deng et al. 2007) by blasting the coding sequence of the first exon of the *Betta splendens* orthologue of each gene. The Integrative Genomics Viewer (Robinson et al. 2011) was used to retrieve the desired region from each genome. Sequences of each gene were aligned in *seaview* (Gouy et al. 2010) using the muscle alignment algorithm and alignments were refined by hand afterward. After alignment, intronic regions were removed for all further analysis if not stated differently.

### *Betta imbellis* Genome Sequencing and Chromosome Assembly, and Gene Annotation

The chromosome-level genome assembly of *B. imbellis* was sequenced and assembled at the Max Planck Institute of Molecular Cell Biology and Genetics in Dresden and was uploaded to NCBI (Assembly Accession Number: PRJNA1098452). Genome sequencing was performed following the protocols of the Vertebrate Genome Project (https://vertebrategenomesproject.org/) (Rhie et al. 2021). High-quality genome assemblies were achieved by combining 35-fold of PacBio HiFi long reads (N50 11,77 kb) and 249-fold of Hi-C Illumina read pairs from a male peaceful betta (*Betta imbillis*). The assemblies were generated using pipelines from the international Vertebrate Genome Project that incorporate state-of-the-art sequencing technologies and assembly algorithms (Rhie et al. 2021). First, we assembled the PacBio HiFi reads into contigs using HiCanu v2.1 (Nurk et al. 2020) and purged retained haplotigs using purge-dups v1.2.3 (Guan et al. 2020). Next, we created scaffolds using Hi-C reads by mapping the reads to the contigs and scaffolding with Salsa2 v2.2 (Ghurye et al. 2019), following the VGP Arima Mapping Pipeline (https://github.com/VGP/vgp-assembly/blob/master/pipeline/salsa/arima_mapping_pipeline.sh). Finally, we performed manual curation on the scaffolds by visualizing the Hi-C contact matrix with HiGlass, removing any falsely incorporated sequences from chromosomes, and creating joins that were missed during the automated scaffolding to produce the final chromosome-scale assembly. The final assembly resulted in 77 scaffolds, with a contig N50 of 21 Mb and a total size of 443.43 Mb. The assembly is at the chromosome level and 99.2% of the assembly is scaffolded into the 21 chromosomes for this species (Fig. S1). The chromosome number is consistent with the karyotype described for *Betta splendens* (Grazyna et al. 2008).

### Estimating Species Relationships

**Fig. 1:**
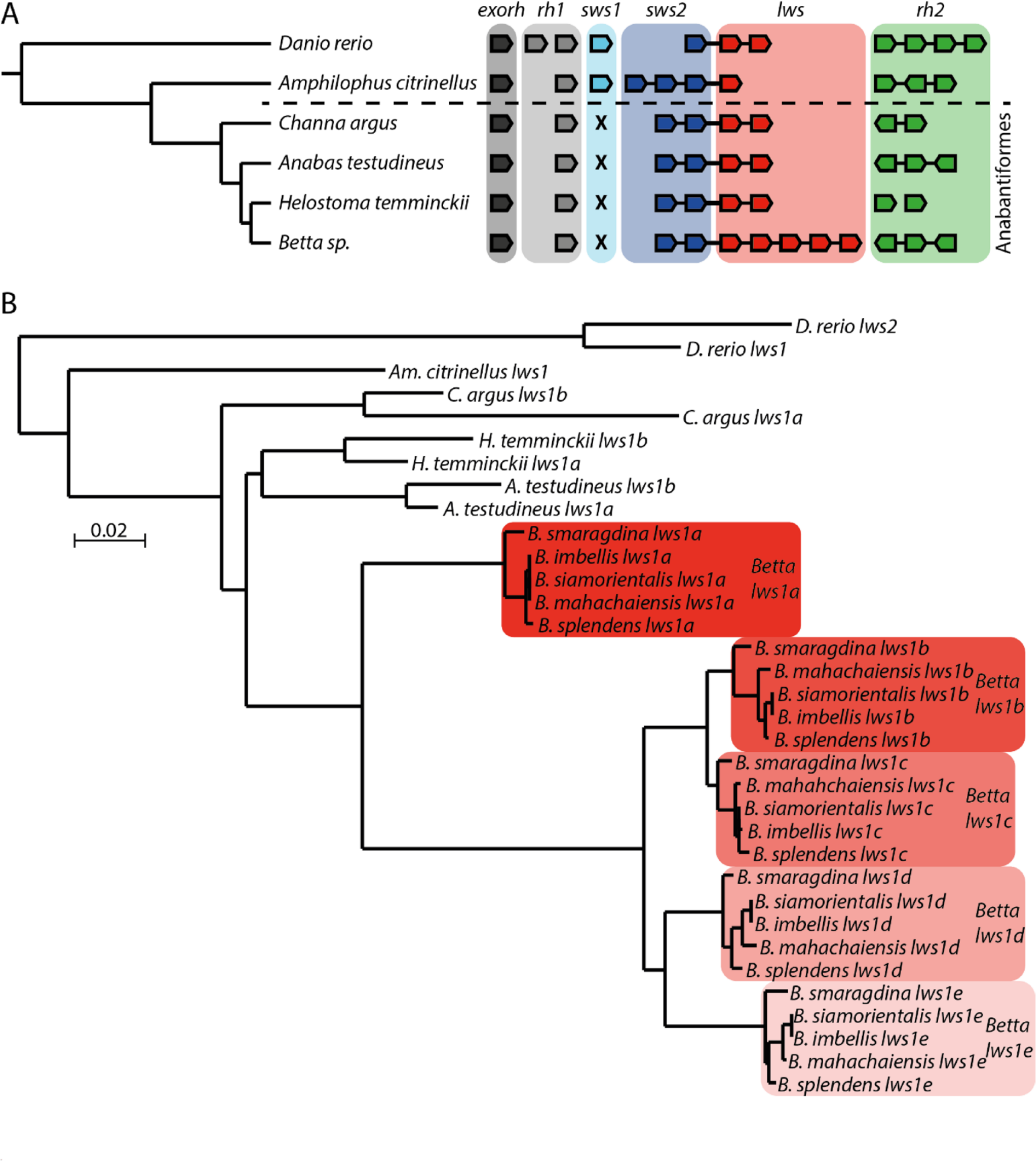
Evolutionary history of opsin genes in the Anabantiformes. **A** A cladogram depicting the relationship of the anabantiform species and outgroups used in this study with illustration of opsin genes found in the genome of each taxon. Species of the genus *Betta* are depicted as a single taxon as there is no evidence of opsin gene number variation within the genus. Physical linkage among opsin genes is depicted using connecting lines. The orientation of the opsin genes to neighboring genes is shown by the shape of the illustrated genes in a 5’—3’ direction. **B** A phylogenetic reconstruction of the coding sequences of all *lws* paralogs suggests that *lws* gene expansion occurred in a common ancestor of the genus *Betta*

The species phylogeny presented in Fig. 1 was created using Orthofinder (Emms and Kelly 2019). Orthofinder uses proteomes to infer species relationships. Coding sequences were collected from ensembl (*B. splendens*, *D. rerio*, *Am. Citrinellus,* and *A. testudineus*) or created by aligning short reads to the reference genome of *B. splendens* and then extracting coding regions using *gffread* and the *B. splendens* annotation (*H. temminckii*, *B. imbellis*, *B. mahachaiensis*, *B. smaragdina,* and *B. siamorientalis*). The phylogeny was estimated using the default settings, except that the multiple sequence alignments (-M msa) option (Emms and Kelly 2019) was used instead of inferring it from the orthogroups.

### Estimating the Evolutionary History of Opsin Genes

Gene trees estimating the relationships of the different opsin genes were generated using IQ-TREE 2 (Minh et al. 2020). Aligned coding sequences were used for the automated model selection (Kalyaanamoorthy et al. 2017) and 1000 bootstraps were performed for each gene tree. We searched for potential breakpoints in the opsin gene alignments indicative of recombination or gene conversion using the Genetic Algorithm for Recombination Detection (GARD) (Kosakovsky Pond et al. 2006) on datamonkey (www.datamonkey.org). The analysis was conducted at the gene class level with the following settings: Run mode: faster; Genetic Code: Universal code; Site-to-site rate variation: General discrete, Rate classes: 4. The synteny of the intergenic regions of opsin genes was analyzed using *R*. Sequence data was handled using the *seqinr* package. Sequences were compared in a sliding window of 25bp using a step size of 3bp using a custom script. Regions that are more than 65% identical appear as a data point in the synteny dot plot.

### Amino Acid Identity

To determine the pattern of opsin sequence divergence between (a) opsin genes in the genus *Betta* and orthologs in outgroup species and (b) among paralogs within the genus *Betta*, we estimated pairwise amino acid distance using a sliding window analysis (30-residue window) following Rennison et al. (2012). In short, we did a series of pairwise comparisons between two sequences: one was the focal opsin gene in the genus *Betta* and the second was an ortholog opsin from an outgroup species (see table S1 for the list of orthologs in outgroup species). This pairwise comparison was repeated for each of the opsin paralogs in each of the five studied species in the genus *Betta*. We report the average amino acid distance from the focal *Betta* opsin gene to each of the orthologs of the outgroup species. This analysis was then repeated to determine the average amino acid distance among paralogs within the genus *Betta*, using the five studied species within the genus as replicates.

Additionally, we tested if *lws* paralogs in the genus *Betta* are evolving under positive or purifying selection (e.g., suggestive of neofunctionalization) or neutral selection (e.g., suggestive of redundant genes) (Lynch and Force 2000). For this, we used codon-based models in PAML (Yang 2007). Different random site models (e.g., M0, M1a, M2a) were compared using log-likelihood ratio tests (LRT) to test for the presence of site classes that differ in dN/dS ratio. Specifically, we first tested for evidence of two site classes (i.e., M1a/M0), one assumed to evolve under purifying selection (dN/dS < 1) and a second class evolving under neutral selection (dN/dS = 1). Second, we tested for the presence of positively selected sites (i.e., M2a/M1a) (Yang 2014).

### Fish Husbandry and Nanopore Sequencing

To determine opsin expression during ontogeny, a single brood of *Betta splendens* was incubated in a large petri dish at 28 °C in a 12:12 dark:light cycle. Three days post-fertilization (dpf), the eggs hatched and the free-swimming larvae were moved to a 500 mL plastic container. At 4 dpf, they were moved to aerated 2 L plastic containers, and at 25 dpf, they were moved to 16 L tanks in a flow-through water system. Larvae were fed infusoria starting at 4 dpf and freshly hatched artemia starting at 6 dpf. Fish were fed frozen adult artemia and red mosquito larvae starting about 8 weeks after fertilization.

For tissue collection, fish were euthanized using tricaine mesylate (MS222). At 3 dpf, 7 dpf, 16 dpf, 20 dpf, 30 dpf, and 40 dpf, whole individuals were euthanized and transferred to RNAlater, stored at room temperature for one hour, and then stored at 4 °C until further processing. For juveniles (120 dpf) and adult (270 dpf), the eyes were dissected and transferred to RNAlater, stored at room temperature for one hour, and then stored at 4 °C until further processing. Individuals from 40 dpf and younger were full siblings. Juveniles and adults were derived from the same breeding stock.

To determine the developmental pattern of opsin gene expression in *B. splendens*, RNA was isolated from whole eye tissue from individuals at each sampled developmental stage. Because isolation from eyes of individuals from the first two developmental stages (3 dpf and 7 dpf) did not yield enough RNA for sequencing, we pooled two heads per developmental stage for an increased yield during RNA isolation. RNA was extracted using TRIzol (ambion©) reagents following the standard trizol-chloroform protocol (Rio et al. 2010). The isolated RNA was prepared for Nanopore long-read sequencing using the PCR-cDNA barcoding kit (SQK-PCB109, Oxford Nanopore Technologies) following the manufacturer’s instructions. Briefly, we used total (non-poly-A selected) RNA for reverse transcription followed by PCR (17 amplification cycles) and barcoding of the samples. After pooling, the 12 samples were sequenced using a single MinION Flow Cell (R9, FLO-MIN106D, Oxford Nanopore Technologies). We chose long-read sequencing over short-read sequencing because the latter could lead to increased mapping errors due to the high amino acid identity between the different *lws* paralogs. An example of this is the annotation of the *B. splendens* genome in the *ensembl* genome browser, where several transcripts span the different *lws* paralogs.

### Nanopore Read Processing and Expression Analysis

After demultiplexing, we used Pychopper (v.2.5.0) for identification, orientation, trimming, and filtering (-Q 10) of full-length Nanopore reads. Reads shorter than 1000 bp were discarded as these were frequently mapped to multiple locations in the genome. We used the nanopore-res-isoforms pipeline to align the remaining reads to the *B. splendens* reference genome. Alignments were visually inspected and the number of reads aligned to each opsin gene was determined using the Integrative Genomics Viewer (Robinson et al. 2011). The proportional expression of each cone opsin gene was calculated as the expression of the target gene divided by the cumulative expression of all cone genes at a given developmental stage (Harer et al. 2018).

## Results

### Opsin Genes in Anabantiformes

In all the anabantoid species analyzed, we identified one copy of the extra-ocular rhodopsin (*exorh*) and one copy of the retinal rhodopsin (*rh1*, Fig. 1A). The cone opsin gene repertoire was found to be more variable (Fig. 1A). All anabantoid species have lost the UV-sensitive opsin *sws1* and have two paralogs of the blue-sensitive opsin *sws2*. Two copies of the green-sensitive opsin *rh2* were identified in the genomes of *C. argus* and *H. temminckii*, but three *rh2* paralogs were found in *A. testudineus* and all the *Betta* species. The highest variation in paralog number was found for the red-sensitive gene *lws*: while in the non-*Betta* species, only two *lws* copies were identified, the genomes of all *Betta* species contain five paralogs of this opsin gene. We did not find copy number variation for any opsin gene among the different *Betta* species (Fig. 1A).

### Evolutionary History of *lws*

The five *lws* paralogs in fish of the genus *Betta* form a single clade, with orthologous genes across the five *Betta* species being more closely related to each other than to paralogs within each species (Fig. 1B), suggesting that the increase in copy number occurred in the common ancestor of *Betta*. On the contrary, the two *lws* paralogs present in *C. argus*, *A. testudineus*, and *H. temminckii* are more closely related to each other than they are to potential orthologs in other species (Fig. 1B). The tree in Fig. 1B suggests independent *lws* duplication events in every anabantoid lineage sampled, including *C. argus*, *A. testudineus*, *H. temminckii,* and *Betta*. An alternative and more likely hypothesis would be to assume a single duplication event in the ancestor of Anabantiformes and subsequent duplication events in *Betta*. In that case, gene conversion among paralogs in each of these anabantoid species could have resulted in more similar *lws* sequences within than between species.

**Fig. 2:**
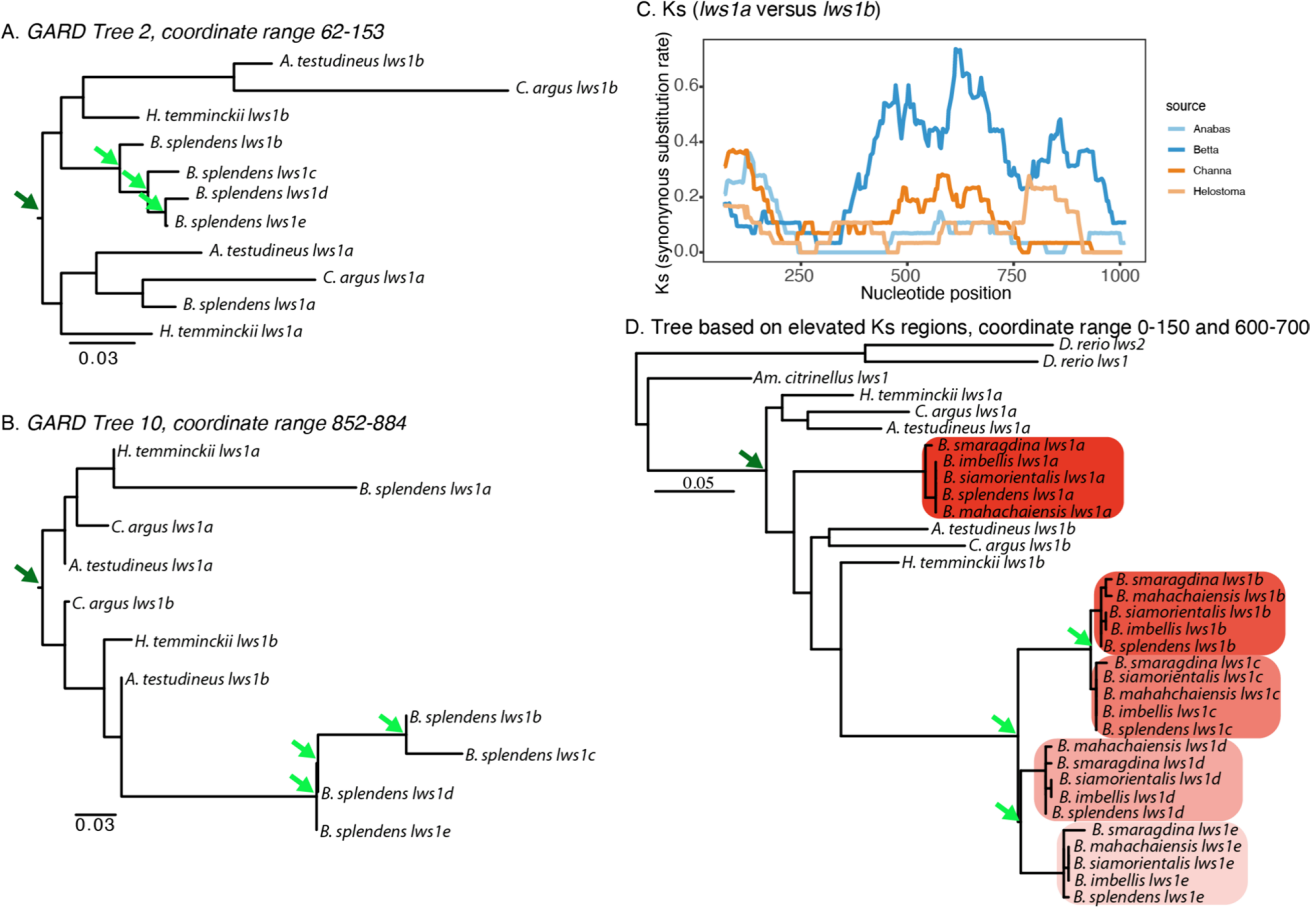
Gene conversion masks the evolutionary relationship of *lws* paralogs in Abantiformes. **A** and **B** Two trees constructed from two fragments bound by the breakpoints identified by GARD show a topology consistent with a common duplication event in the ancestor of all anabantoid fishes (dark green arrow), followed by gene conversion and at least two, possibly three duplication events in the genus *Betta* (light green arrows). **C** Pairwise synonymous substitution rates (Ks) between *lws1a* and *lws1b* in four genera of anabantoid fishes. Decreases in Ks to zero in different regions of the coding sequence of *lws1* paralogs suggest gene conversion. **D** A phylogenetic reconstruction based only on fragments of the coding region where Ks is relatively high in all species (concatenation of 0–150 and 600–700 bp) is also consistent with a single duplication event in the ancestor of Anabantiformes (dark green arrow) followed by three duplications in the genus *Betta* (light green arrows) (Color figure online)

Recombination breakpoint analysis in GARD (Kosakovsky Pond et al. 2006) and pairwise sliding window analysis of neutral divergence (synonymous substitution rate, Ks) in DnaSP V.6 (Rozas et al. 2017) supported the hypothesis of a single duplication event in the common ancestor of the four anabantoid species studied, followed by additional duplication events in the common ancestor of *Betta*. GARD identified ten putative recombination breakpoints in the anabantoid *lws* alignment (i.e., Fig. 1B without outgroups), suggesting that gene conversion might have occurred multiple times among *lws* paralogs. A model that allowed for different topologies between fragments fit the data better than one that assumed the same tree across fragments (ΔAIC = 293.248). Two of the resulting topologies support the hypothesis of a common duplication event (Fig. 2). Sliding window analysis of neutral variation further suggests that gene conversion affects *lws* paralogs in all species examined. Pairwise comparisons showed that the rate of synonymous substitutions between *lws1a* and *lws1b* within species decreases significantly along different parts of the coding region (Fig. 2). A phylogeny based only on the fragments of the coding region where all species have relatively high Ks (0–150 and 600–700, Fig. 2) also yields a topology congruent with one duplication event in the common ancestor of anabantoids, followed by three duplication events in *Betta*.

**Fig. 3:**
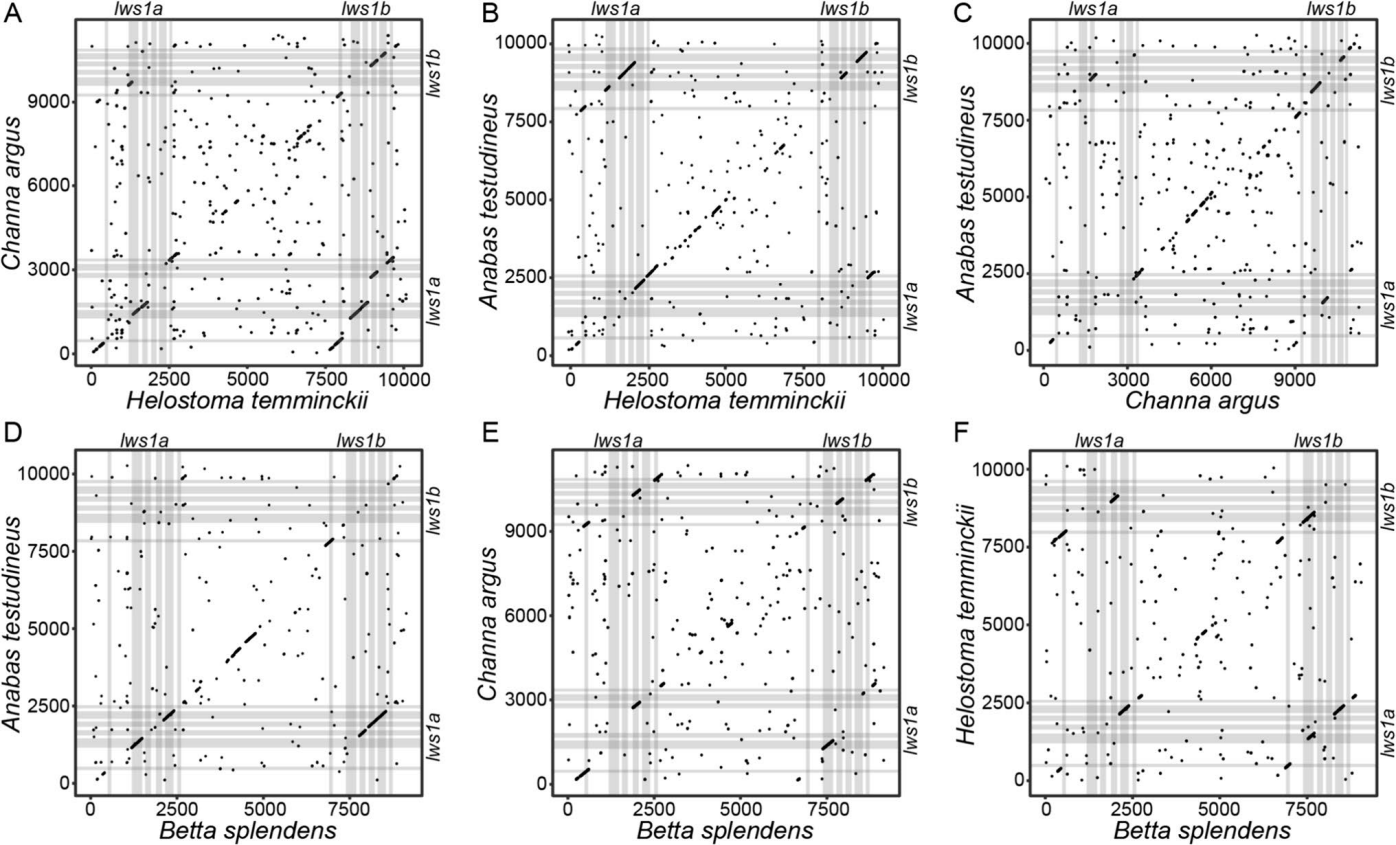
Synteny dot plots of genomic region harboring *lws1a* and *lws1b*. Synteny between **A** *C. argus* and *H. temminckii*, **B** *H. temminckii* and *A. testudineus*, **C** *C. argus* and *A. testudineus*, **D** *B. splendens* and *A. testudineus*, **E** *B. splendens* and *C. argus*, and **F** *B. splendens* and *H. temminckii*. Each black dot indicates a 25 bp window having a sequence identity of 65% or higher between the two compared genomes. Gray boxes indicate the positions of exons of the two *lws* paralogs

To further explore if the two *lws* paralogs found in each of the early diverging anabantoid species (*C. argus*, *H. temminckii,* and *A. testudineus*) originated through independent duplication events, we compared the intergenic region between the *lws* paralogs in a syntenic dot plot. Pairwise comparisons between *C. argus*, *H. temminckii, A. testudineus,* and *B. splendens* revealed substantial preservation in the genomic architecture of the region between *lws1a* and *lws1b* in all pairwise comparisons. The distance between *lws1a* and *lws1b* ranges from 5 to 6 kb in all four species. There is some, but not substantial nucleotide conservation among the species of this region (Fig. 3), likely reflecting the long divergence time. Nonetheless, in almost all cases, there is clear evidence that some of the coding sequence fragments in one paralog are very similar to segments in both paralogs of the other species (e.g., Fig. 3A), providing further support to the hypothesis of common origin followed by gene conversion. In some cases, parts of the intergenic region are more similar than the protein-coding sequences (e.g., Fig. 3B), again supporting a single origin. Taken together, these results favor the hypothesis of a single duplication event in the common ancestor of Anabantiformes that resulted in the presence of *lws1a* and *lws1b* paralogs in the different species followed by gene conversion.

### Evolutionary History of *rh2 *and *sws2*

Similar to *lws*, the analysis of the coding sequences of *rh2* resulted in a gene tree mainly reflecting the species phylogeny, except among species of the genus *Betta*. In the genus Betta, the three orthologous genes clustered together (Fig. S2A). Outside the genus *Betta*, the different *rh2* paralogs clustered according to species, again suggesting independent duplication events in the different lineages or high levels of gene conversion (Fig. S2A). The analysis with GARD revealed two breakpoints indicating possible recombination events between the different paralogs. However, phylogenies based on the single gene segments between breakpoints did not change the phylogenetic relationship among *rh2* paralogs (Fig. S3).

The analysis of the *sws2* sequences resulted in a phylogeny that does not reflect the species tree but the evolutionary history of the genes. *sws2a* and *sws2b* clustered into two distinct groups and within each of those groups, the genes reflected the species phylogeny. This indicates that the presence of two *sws2* paralogs in the Anabantiformes results from an ancient gene duplication in a common ancestor of cichlids and Anabantiformes (Fig. S2B).

### Amino Acid Substitutions and Spectral Sensitivity

**Fig. 4:**
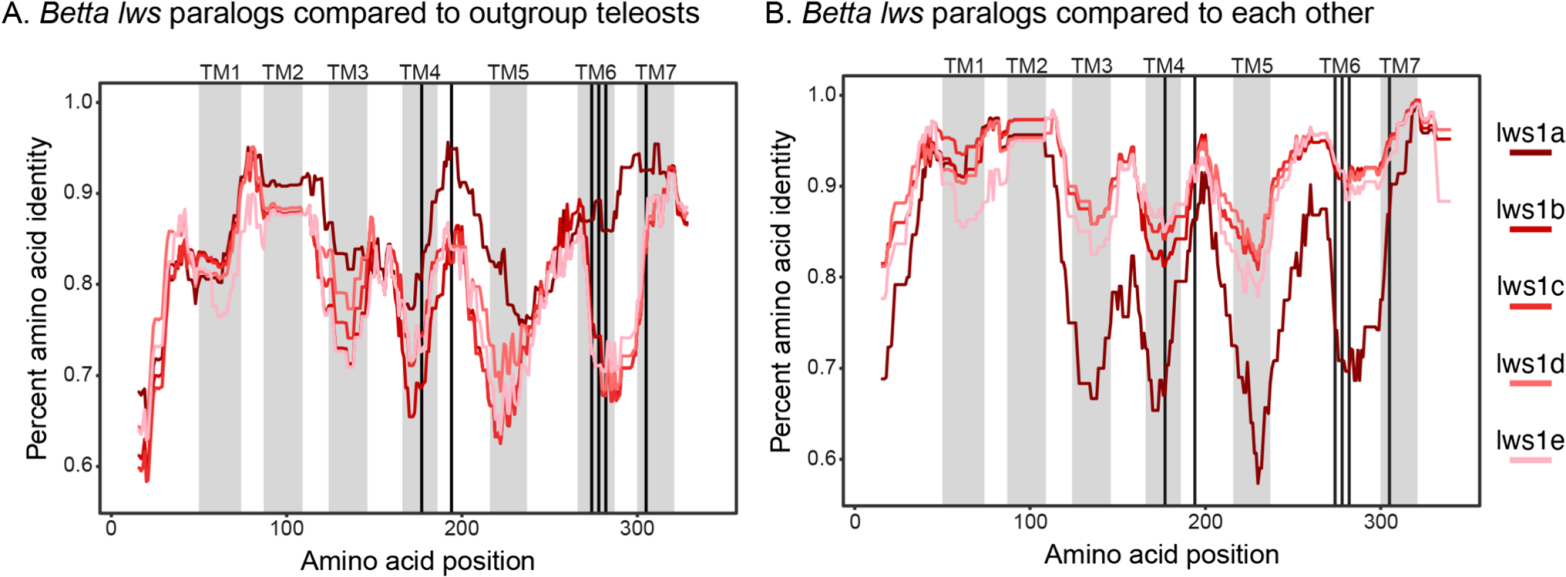
Sliding window analysis of LWS paralogs amino acid sequence identity. Each red line represents **A** the mean divergence between one LWS paralog of all *Betta* species and LWS sequences of a set of representative teleost species (see table S2) or **B** the mean divergence between one LWS paralog of all *Betta* species and the remaining four paralogs in *Betta*. Gray areas indicate the position of the seven transmembrane domains of LWS (labeled on top from TM1–TM7). Dark vertical bars indicate the positions of LWS key-tuning sites (based on Yokoyama et al. 2008b) (Color figure online)

Consistent with nucleotide-level results (Fig. 1), our amino acid-level analyses revealed divergence within LWS and RH2 that cannot be attributed to ancestral differentiation and thus appear novel for the genus *Betta*. All LWS paralogs in the genus *Betta* show significant divergence from LWS orthologs in the outgroup set (see table S1 for the list of orthologs in outgroup species). Congruent with the phylogenetic reconstruction at the nucleotide level (i.e., Fig. 1), LWS1b, LWS1c, LWS1d, and LWS1e are the more divergent from LWS in the outgroups. However, LWS1*a,* which shows the least divergence, still has an average amino acid sequence identity below 85% (Fig. 4). All three copies of RH2 have diverged to a similar degree from the RH2 ortholog in other teleost species (Fig. S4). Differences in the two paralogs of SWS2 appear to reflect similar divergence as seen in other teleost species (Fig. S5). Our analyses of the coding sequence evolution of the five *Betta* LWS paralogs suggest that this divergence is not due to neutral evolution, but that these genes are evolving under purifying selection (Table S3).

Sliding window analysis of the LWS amino acid sequence revealed high rates of amino acid substitutions between *Betta* and outgroup species in four distinct regions corresponding to four of the seven transmembrane domains of the opsin protein (i.e., transmembrane domains III, IV, V, and, Fig. 4) VI (Carleton et al. 2005; Chang et al. 1995). The only exception is *lws1a*, which shares a relatively high amino acid identity with *lws* orthologs in outgroup species at transmembrane domains III and VI. Overall, *lws1a* is the paralog most similar to other teleost *lws* genes. The remaining four paralogs diverged similarly from the outgroup *lws* sequence (Fig. 3A) and *Betta lws1a* (Fig. 3B). Within species of the genus *Betta*, the *lws* paralogs diverged most in four regions overlapping transmembrane domains III to VI (Fig. 3B), similar to the pattern seen when compared with outgroup species (Fig. 3A). This pattern is not driven by *lws1a* alone, as most paralogs diverged to some degree in these transmembrane domains (see Fig. S6). Two of these regions of differentiation are close to three of the five key-tuning sites that are important for spectral tuning of *lws* genes (Asenjo et al. 1994; Yokoyama and Radlwimmer 2001).

The sliding window analysis of the amino acid sequences of RH2 revealed three regions of differentiation between the paralogs of the genus *Betta* and the RH2 of other teleost species (Fig. S4A). Only two of those are in the transmembrane regions of the protein and one is located at one of the extracellular domains of the protein. All three paralogs show similar levels of differentiation from other teleost’s *rh2* paralogs (Fig. S4A). The within comparison of *Betta* paralogs showed only one region of strong differentiation corresponding to one of the areas found in the within teleost comparison (Fig. S4B). The loss of the other two regions of differentiation in the within *Betta* comparison indicates that the differentiation in these two regions is exclusive to *Betta* and not just a signal due to inter-paralog differences (Fig. S4B).

The comparison of SWS2 within teleost revealed two regions with high rates of amino acid substitutions, one of them located at the second extracellular domain, and the second one located at the fourth transmembrane of the protein. The within *Betta* comparison shows a similar pattern of differentiation between the two paralogs in *Betta*, indicating the differences found in the teleost-wide comparison just reflect the general differences between the two paralogs that can be found in most teleost species (Fig. S5).

While it is not possible to determine the spectral sensitivity of each of the opsin paralogs from the amino acid sequence, amino acid substitutions at certain key sites of each opsin class allow us to estimate relative changes of the maximum spectral sensitivity between different paralogs of the same opsin class (Chi et al. 2020; Yokoyama and Jia 2020; Yokoyama and Radlwimmer 1998, 2001). Two of the five key sites to tune the spectral sensitivity of *lws* show variation between paralogs in all analyzed *Betta* species. A shift from serine (S) to alanine (A) at residue 177 (residue 164 in bovine rhodopsin) (S177A) in *lws1a* and *lws1b* is expected to shift the maximum sensitivity of the resulting visual pigment toward shorter wavelengths compared to the sensitivity of visual pigments derived from the other three *Betta* paralogs and to that seen in the three early diverging genera of Anabantiformes (Asenjo et al. 1994; Yokoyama and Radlwimmer 2001). The exemption to this was *lws1b* in *B. smaragdina*, which showed S177. The substitution Y274F (261 in bovine rhodopsin) seen in *lws1a* for all *Betta* species is expected to further shift the sensitivity of the resulting visual pigment toward shorter wavelengths. These two amino acid substitutions suggest a short-wavelength shifted λ_max_ for *lws1a*, an intermediate λ_max_ for *lws1b,* and a long-wavelength shifted λ_max_ for the remaining three paralogs (Table 1). The substitution E130Q (122 in bovine rhodopsin) at the known key-tuning site in *rh2* indicates a shift of *rh2aγ* λ_max_ toward longer wavelengths, too (Imai et al. 1997; Yokoyama and Jia 2020). The three key-tuning sites of the *sws2* gene (Cortesi et al. 2015; Yokoyama 2008) did not show any variation between paralogs and species.

**Table 1:** Estimated maximal absorption of *lws* opsin genes in *Betta splendens* based on amino acid substitutions at sites of the five key-tuning sites of the “five-sites” rule (Yokoyama et al. 2008a, b)

Tuning site		Ancestral	*Betta* paralogs*				
*Bo. taurus*	*B. splendens*	*lws*	*lws1a*	*lws1b*	*lws1c*	*lws1d*	*lws1e*
164	177	S	A	A*	S	S	S
181	194	H	H	H	H	H	H
261	274	Y	F	Y	Y	Y	Y
269	282	T	T	T	T	T	T
292	305	A	A	A	A	A	A
Estimated λ_max_ (nm)		560	545	555	560	560	560

The ancestral *lws* sequence was inferred as the consensus sequence from the three early diverging genera of Anabantiformes included in this study

*Except for *B. smaragdina*, which showed S177, all five species of the genus Betta were invariant in these five residues

### Cone Opsin Expression During Ontogeny of *Betta splendens*

**Fig. 5:**
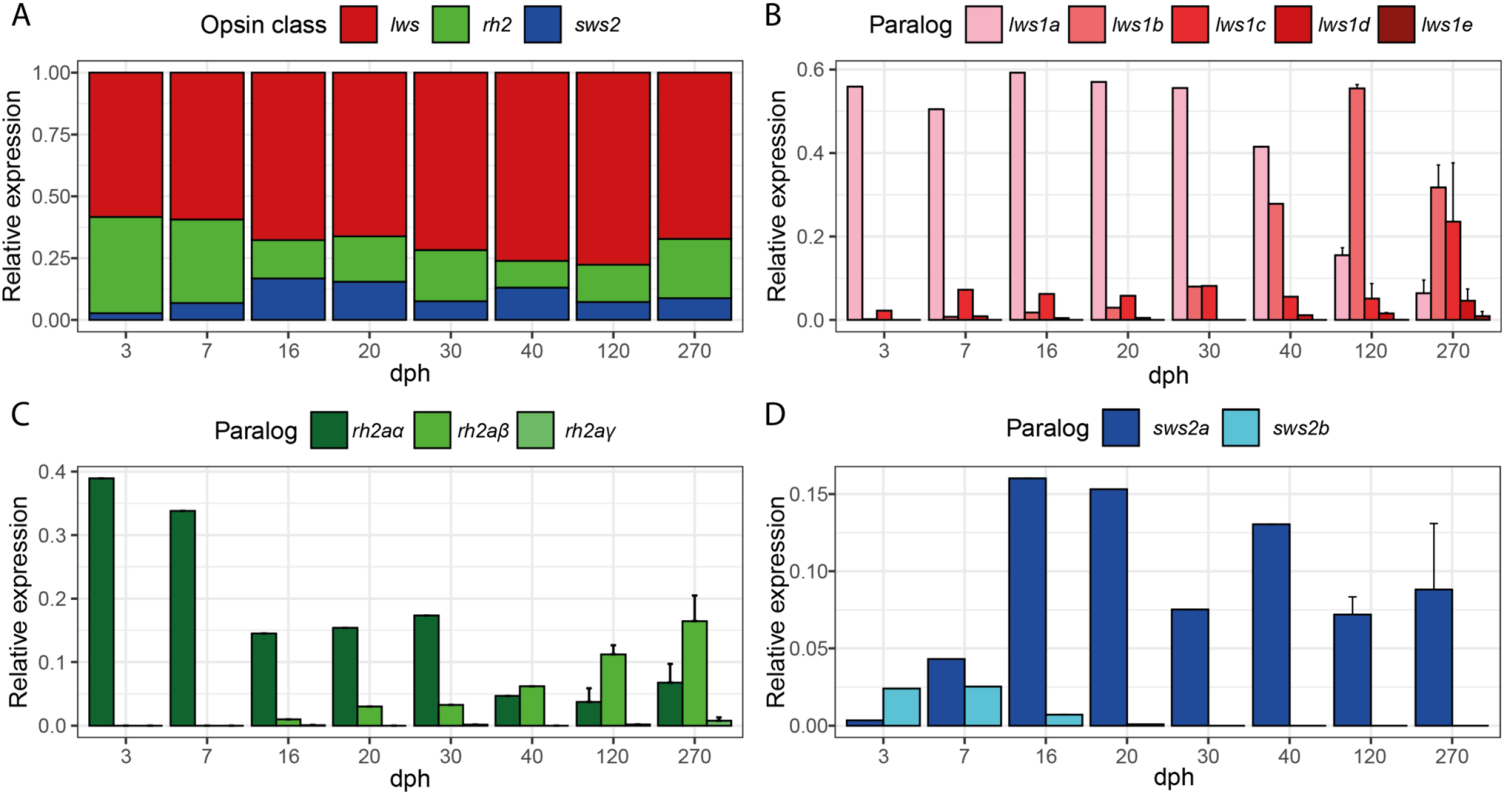
Ontogenetic change of relative expression of different opsin paralogs in *B. splendens*. **A** Stacked bars plot showing the sum of relative expression of all paralogs of each opsin class at each of the eight studied ontogenetic stages. Bars in panels **B**–**D** represent the relative expression of each paralog of *lws* (**B**), *rh2a* (**C**), and *sws2* (**D**) at each studied ontogenetic stage. Error bars at stages 120 and 270 indicate standard deviation. dpf = days post-fertilization

At all developmental stages, the cumulative expression of the five *lws* paralogs constituted more than half of the total cone opsin gene expression in *B. splendens*. The highest proportional expression of the *Rh2* paralogs occurred early in development but decreased later in life. The proportional expression of *sws2* was constantly low, peaking at 16 dpf to 40 dpf when it accounted for around 15% of total cone opsin expression (Fig. 5D). Our analysis shows that the 10 cone opsin genes found in the genome of *B. splendens* were expressed across ontogeny. Expression repertoires differ between early and later developmental stages (Fig. 5). Generally, paralogs of the different opsins expressed at early developmental stages were replaced by the expression of a more diverse set of paralogs later in development. Adult individuals expressed nine of the ten different cone opsin genes at the same time, with at least seven of those accounting for at least 5% of the total cone opsin gene expression (Fig. 5).

Among *lws* paralogs, *lws1a* was the dominant opsin until 40 dpf, accounting for 40–60% of the total cone opsin expression (Fig. 5). Expression of *lws1a* decreased in juvenile and adult individuals but did not fully cease (Fig. 5C). *lws1b* started to be expressed at low levels from 7 dpf on, considerably increasing expression at 40 dpf and replacing *lws1a* as the most highly expressed gene in juveniles and adults. *lws1c* was expressed at low levels from 3 dpf on and showed increased expression in adults only. The other two paralogs, *lws1d* and *lws1e*, generally show low proportional expression levels. Expression of *lws1d* could be detected at all stages except at 3 dpf and 30 dpf and it was the highest in adults. *lws1e* showed low expression levels only in juveniles and adults. Except for *lws1a*, all *lws* paralogs showed increased expression toward the later stages of life. Adults expressed all five paralogs of *lws* simultaneously, with only *lws1e* constituting less than 5% of total cone opsin expression (Fig. 4C).

Ontogenetic changes in expression were also seen for the green wavelength-sensitive *rh2* opsin genes (Fig. 5B). *rh2aα* showed high proportional expression levels at 3 dpf and 7 dpf and then decreased its expression steadily toward adulthood. Starting at 16 dpf, the expression levels of *rh2aβ* increased, replacing *rh2aα* as the *rh2* paralog with the highest expression at 40 dpf and further increasing its relative expression in juveniles and adults. *rh2aγ* showed almost undetectable levels of expression during some stages (16 dpf, 30 dpf, 90 dpf) and low levels of expression in adults. As already seen for *lws* paralogs, adults expressed all different paralogs of *rh2* at the same time, although *rh2aγ* is expressed at low levels (Fig. 5B).

The expression patterns of the blue-sensitive opsins differ from other opsins in *B. splendens* (Fig. 5C). Both paralogs, *sws2a* and *sws2b,* are expressed at 3 dpf. While the proportional expression of *sws2a* increased until 16 dpf and stayed constant until adulthood, the proportional expression of *sws2b* decreased through time and became silent at 30 dpf. Contrary to *lws* and *rh2*, both paralogs of *sws2* are expressed at the same time during early ontogenetic stages and only one of them is expressed during adulthood (Fig. 5C).

## Discussion

In this study, we investigated the evolution of visual opsins in the teleost order Anabantiformes focusing on the evolution of the cone opsin gene *lws* in species of the genus *Betta*. The analysis of the whole-genome sequences of eight anabantoid species revealed the loss of *sws1* in the entire order and the expansion of the *lws* gene family to five copies in species of the genus *Betta*. Further analysis of amino acid sequences in the genus *Betta* and gene expression throughout ontogeny in *B. splendens* suggests functional diversification of the different *lws* paralogs, possibly driven by temporal collinearity in their expression patterns.

### Evolution of Cone Opsin Gene Number in Anabantiformes

Our search for opsin genes in the genomes of eight species of Anabantiformes revealed both losses and duplications of different opsin genes. The complete loss of *sws1* genes (Lin et al. 2017; Musilova et al. 2019) or the reduction or loss of *sws1* expression in adults (Escobar-Camacho et al. 2017; Hofmann et al. 2009; Musilova and Cortesi 2021; Torres-Dowdall et al. 2021) is common in teleost fishes occurring in aquatic habitats and has been described for multiple freshwater teleost species (Escobar-Camacho et al. 2020; Hauser et al. 2021; Liu et al. 2019). Oxidative stress after absorption of UV light can result in retinal damage (Ivanov et al. 2018), selecting for UV-filtering lenses, as shown for the anabantoid species *H. temminckii* and *Trichopodus leeri* (Douglas and McGuigan 1989). Once UV-filtering lenses evolve, UV-sensitive pigments based on *sws1* are rendered obsolete and often pseudogenized (e.g., Weadick et al. 2012). Also, the strong scattering of UV light by particles in water might be a disadvantage as the glaring effect might blind the fish. Eventual losses of spectral sensitivity in the non-UV low wavelengths through the loss of *sws1* could be compensated by changes in spectral sensitivity of *sws2* paralogs, particularly at early stages of development (reviewed in Lupše et al. 2022). However, the proportional expression of *sws2* paralogs does not vary significantly across ontogeny in *B. splendens* (Fig. 5A).

We did not find variation across species in the number of *sws2* paralogs, as all the investigated anabantoid species possess two copies of this gene (Fig. 1). This is in line with previous findings suggesting that *sws2* underwent one tandem duplication in the ancestor of Neoteleostei and a second duplication (of *sws2a*) in the ancestor of Percomorpha (Cortesi et al. 2015; Rennison et al. 2012). None of the studied species has two *sws2a* paralogs, suggesting that this gene was lost early in the evolution of Anabantiformes.

The green wavelength-sensitive opsin *rh2* is the opsin that shows the highest variation in copy number along the teleost phylogeny, ranging from zero to eight paralogs (Musilova and Cortesi 2021). High numbers of *rh2* paralogs are mainly found in marine species inhabiting the open ocean (de Busserolles et al. 2020). Contrary to the high variation found in other lineages (Musilova and Cortesi 2021), we find little variation of *rh2* copy number within the Anabantiformes. While *C. argus* possesses two *rh2* copies, the genomes of *A. testudineus* and the five *Betta* species contain three *rh2* paralogs. We found two *rh2* copies in the genome of *H. temminckii*, both of which were on the border of two different scaffolds preventing a reliable estimation of *rh2* copy numbers in that species due to low genome assembly quality.

In contrast to the other cone opsin genes, the long-wavelength-sensitive gene *lws* experienced several duplication events within the anabantoid lineage. Whereas the genomes of *C. argus*, *H. temminckii* and *A. testudineus* contain two copies of *lws*, five paralogs were found in the genomes of all five studied *Betta* species. Most teleost species possess only one or two copies of *lws* (Musilova et al. 2019), except for a handful of lineages that experienced exceptional duplication of *lws* leading to up to five copies in some species, including *B. splendens* (Cortesi et al. 2021). In some rare cases, the presence of multiple *lws* copies results from ancient teleost-specific genome duplications, but most species attained their additional paralogs through relatively recent tandem duplications (Cortesi et al. 2021). Although the gene tree suggests independent duplication events of *lws* in each lineage (*Channa*, *Helostoma*, *Anabas,* and *Betta*), analyses of recombination, sliding window analyses comparing synonymous substitution rates, and synteny analysis of the region between *lws1a* and *lws1b* suggest a duplication event in the common ancestor of anabantoid fishes, followed by gene conversion within lineages. This means that at least two and possibly three, more recent tandem duplication events of *lws* must have occurred in the lineage leading to *Betta*. This is a slightly different interpretation from that of Cortesi et al. (2021), who suggested one duplication in *H. temminckii* and *A. testudineus* and three duplications in the *Betta* lineage. In general, the high number of *lws* paralogs found in *Betta*, and perhaps the two copies found in other anabantoid species, may reflect the habitats in which many of these species occur. While species of the Anabantiformes occur in a wide range of biotopes, many of them inhabit soft, acidic, and mineral-poor water that is stained by humic substances (black water) (Linke 2014), which might favor a shift of the spectral sensitivity into the red spectrum.

Using phylogenetic reconstructions to understand the evolutionary relationships of opsins proved to be problematic, as gene conversion between opsin paralogs is common (Hiwatashi et al. 2011; Reyniers et al. 1995; Zhao et al. 1998) and can suppress paralog differentiation, but not between species, as potentially seen for *lws*. On the other hand, gene conversion can prevent the pseudogenization of duplicated genes and even recover pseudogenized paralogs (Cortesi et al. 2015; Mighell et al. 2000), retaining copy number variation and diversification between species, but decreasing within species differentiation at the same time.

### Spectral Tuning Through Amino Acid Substitution

The high number of different opsin genes and paralogs of the same opsin class in some teleost species allows for nuanced fine-tuning of visual sensitivity to the photic environment (Carleton et al. 2005). For this to happen, different paralogs of the same opsin class must vary in their spectral sensitivity, which is generally achieved through amino acid substitutions at key residues in the protein sequence of the opsin (Yokoyama 2008).

Our analysis of the amino acid sequence of *lws* paralogs of the different *Betta* species revealed four regions corresponding to four of the seven transmembrane domains of *lws* that seem to have diverged from the *lws* sequence of outgroup species. Phylogenetic methods for detecting molecular evolution found no evidence that the *lws* paralogs in the genus *Betta* evolve under no selection, suggesting instead that purifying selection acts on these paralogs (table S3). *Lws1a* is the *Betta* paralog most similar to *lws* orthologs in outgroup species, while *lws1b, lws1c, lws1d,* and *lws1e* differ from orthologs at the same positions (Fig. 4A). More importantly, when comparing the *lws* paralogs of *Betta* with each other, we see a very similar trend: *lws1a* diverged from the other paralogs at the same position that diverged between *Betta* and outgroup species, while the remaining paralogs show lower levels of divergence (Fig. 4B). Again, the regions of differentiation correspond to four of the transmembrane domains of the opsin protein. This indicates functional differences between *lws1a* and the other paralogs, as the binding pocket of the chromophore is located in the transmembrane domains and hence affects the spectral sensitivity of the resulting visual pigment. More precisely, we found that 10 of the 59 amino acids directed into the binding pocket of the chromophore (Carleton et al. 2005) are variable among the five *lws* paralogs. This pattern of divergence suggests that *lws1a* retained the spectral characteristics of *lws* in the common ancestor of Anabantiformes, and *lws1b, lws1c, lws1d, and lws1e* diverged to maximally absorb photons at different wavelengths.

While, in most cases, it is not possible to make precise predictions about the changes in the wavelength of maximum absorption (λ_max_) of an opsin in response to amino acid substitutions (for exceptions see Patel et al. 2018), there are amino acid substitutions of five key sites of *lws* that have been studied intensively (Yokoyama 2008; Yokoyama et al. 2008b). We found that two of these five positions are variable between the different *lws* paralogs of *Betta*. The amino acid substitutions that we found indicate that λ_max_ of *lws1a* is shorter than the λ_max_ of the remaining paralogs and that the λ_max_ of *lws1b* is shorter than the λ_max_ of *lws1c*, *lws1d,* and *lws1e*. Some of the other amino acid substitutions observed here might affect the λ_max_ of the different paralogs, as four of the five key-tuning sites are located within regions showing high amino acid divergence, and spectral changes due to amino acid substitutions at key-tuning sites can depend on the genetic background (Chinen et al. 2005). Even though we cannot precisely determine the spectral sensitivity of the different *lws* paralogs, our results of overall amino acid divergence and changes at key-tuning sites suggest that there are functional differences between the *lws* paralogs that allow the *Betta* species to maximally absorb photons at different wavelengths.

### Ontogenetic Changes of Opsin Expression

Our analysis of opsin expression at eight different ontogenetic stages from the early larval stages to adulthood of *B. splendens* shows that each opsin gene found in the genome of *B. splendens* is expressed at least at one ontogenetic stage. While the paralogs of all three cone opsin classes show considerable changes in expression, the relative expression between opsin classes remains relatively stable throughout the developmental stages. This indicates that changes in spectral sensitivity between ontogenetic stages are rather achieved by switching between functionally divergent paralogs instead of changing the ratio of opsin classes, both common strategies in teleost fishes (Chang et al. 2021; Harer et al. 2017; Lupše et al. 2022). Paralogs with dominant expression in the early stage are always replaced by other paralogs during later developmental stages. Contrary to many other teleost species, that only express a small subset of cone opsin genes during adulthood (Harer et al. 2017; Spady et al. 2006), we found expression of 9 out of 10 existing cone opsin genes in adults of *B. splendens*, with at least seven of these accounting for at least 5% of the total opsin expression. We acknowledge that this does not necessarily mean that all expressed genes are translated into functional opsin proteins, and future studies will be needed to determine if visual pigments in the retina of *B. splendens* reflect the diversity suggested by opsin gene expression.

The changes in expression levels of different paralogs of *lws* indicate a general shift of the spectral sensitivity from shorter wavelengths in the early developmental stages to longer wavelengths in later developmental stages. Paralogs with the lower λ_max_ are expressed early while paralogs with higher λ_max_ are expressed during later developmental stages. This result is similar to observations in other teleost species where a general trend from short-wavelength sensitivity in early developmental stages to long-wavelength sensitivity in late developmental stages could be observed (Harer et al. 2017; Shand et al. 2008; Spady et al. 2006). This plausibly reflects changes in microhabitat use and diet associated with ontogeny.

### Temporal Collinearity may Drive Neofunctionalization of *lws* Paralogs

The evolution of spectral sensitivities different from the ancestral state (i.e., neofunctionalization) and the evolution of spatial and temporal division of the original function (i.e., subfunctionalization) are common for opsin genes (e.g., Carleton et al. 2008; Chang et al. 2021; Härer et al. 2017; Owens and Rennison 2017; Owens et al. 2011; Rennison et al. 2011; Spady et al. 2006; Torres‐Dowdall et al. 2021; Tsujimura 2020). Here, we observed subfunctionalization in the expression of *lws* paralogs in *B. splendens* across ontogeny, similar to other fish (e.g., Härer et al. 2017; Lupše et al. 2022). Interestingly, paralogs located closer to the 5’ end of the genomic cluster containing the five *lws* paralogs start being expressed early during development, while those closer to the 3’ end start being expressed later. This means that the relative position of the paralog in the genome corresponds to the timing of expression during ontogeny. This phenomenon, called temporal collinearity, is one of the most notable characteristics of the expression of *hox* genes during embryogenesis (Duboule 1994). Although the cause of the temporal shift in *hox* gene expression is unclear, changes in chromatin structure are known to make downstream genes available for transcription in later development stages (Deschamps and Duboule 2017; Noordermeer et al. 2014). Temporal collinearity adds the intriguing possibility that the selection pressures on each paralog might be determined by its position in the *lws* cluster, as their time of expression during ontogeny is dependent on this position. In other words, the pattern of temporal subfunctionalization might influence the pattern of neofunctionalization of spectral sensitivity. In fact, our inferred spectral sensitivity for each paralog (Table 1) matches the common progression from short-wavelength to long-wavelength sensitivity seen across ontogeny in many teleost fishes (Lupše et al. 2022) and varies gradually from the 5’ end to the 3’ end of the genomic cluster. Similar interactions between expression regulation and neofunctionalization in opsin genes are seen in other animals. In zebrafish, the expression of tandemly duplicated opsin paralogs is controlled by a single promoter region, and the relative position of each paralog affects their spatial expression. This spatial pattern is correlated with changes of spectral sensitivity, with variation from short- to long-wavelength shifted paralogs that are expressed along a central dorsal-ventral axis of the retina (Tsujimura 2020; Tsujimura et al. 2007, 2015). In humans, there is copy number variation in the middle-to-long-wavelength-sensitive opsin genes (*opn1lw* + *opn1mw* + (N × *opn1mw*), Neitz et al. 1995). A common locus control region activates the transcription of the two nearest opsins (*opn1lw* and *opn1mw*) in a spatial collinearity pattern, while the rest of the copies are not expressed to significant levels (Cooper et al. 2007). The first paralog in humans retains the ancestral sensitivity to longer wavelengths, whereas the second shows neofunctionalization through mutations that shift its sensitivity toward shorter wavelengths (Asenjo et al. 1994; Nathans et al. 1986). This pattern of opsin expression remains constant during human development, unlike what we observed in *B. splendens*, but it also suggests that subfunctionalization influences neofunctionalization. Although our study does not provide conclusive evidence, it suggests that temporal collinearity might promote the persistence and diversification of *lws* genes in *Betta* and potentially other lineages. This offers a testable hypothesis for the differentiation pattern of opsin genes in teleost fishes.

## Conclusion

The genomic analyses of different anabantoid species revealed that at least two *lws* duplication events occurred in the *Betta* lineage, resulting in the expansion of *lws* copy number in this genus. Differences in amino acid sequences hint at functional differentiation between *lws* paralogs within *Betta*. The analysis of eye transcriptomes revealed an ontogenetic shift of opsin expression and suggested temporal collinearity as a factor driving *lws* sequence diversification in *Betta* fishes.

### Supplementary Information

Below is the link to the electronic supplementary material.

Supplementary file1 (DOCX 2320 kb)
