# Supplementary material for: Expansion and Functional Diversification of Long-Wavelength-Sensitive Opsin in Anabantoid Fishes
Source: J Mol Evol. 2024 Jun 11;92(4):432–48. doi: 10.1007/s00239-024-10181-0 (PMC11291592; doi:10.1007/s00239-024-10181-0)
Supplement: Supplementary file 1 — Supplementary file1 (DOCX 2320 kb) [file 239_2024_10181_MOESM1_ESM.docx]

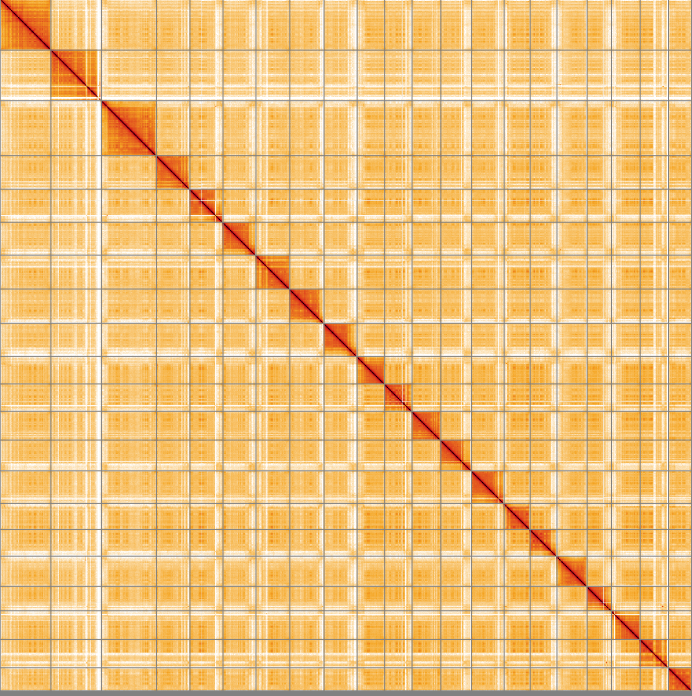


**Figure S1**: Hi-C heat map of intra- and inter-chromosomal (Chr 1-Chr 21) interactions throughout the genome of *Betta imbellis* (NCBI accession number PRJNA1098452) supporting 21 chromosomes. Hi-C map visualized using HiGlass (Kerpedjiev et al. 2018).{Kerpedjiev, 2018 #920}{Kerpedjiev, 2018 #920}


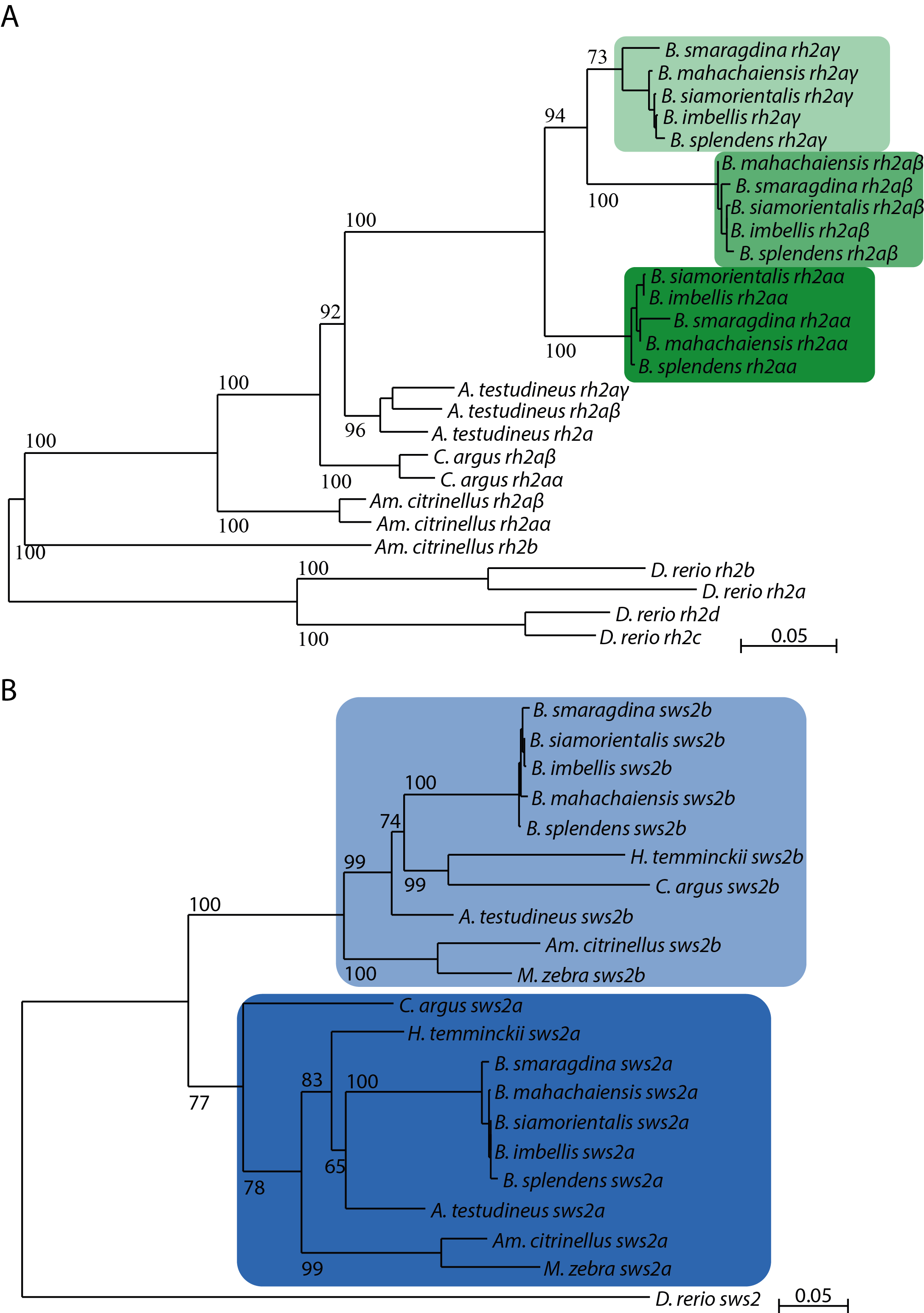


**Figure S2**: Phylogenetic reconstruction of coding sequences of rh2 (A) and sws2 (B). Green boxes indicate clades of *rh2a*, *rh2b* and *rh2c* in *Betta* (A), blue boxes indicate clades of *sws2a* and *sws2b* in all acanthomoprh species (B).


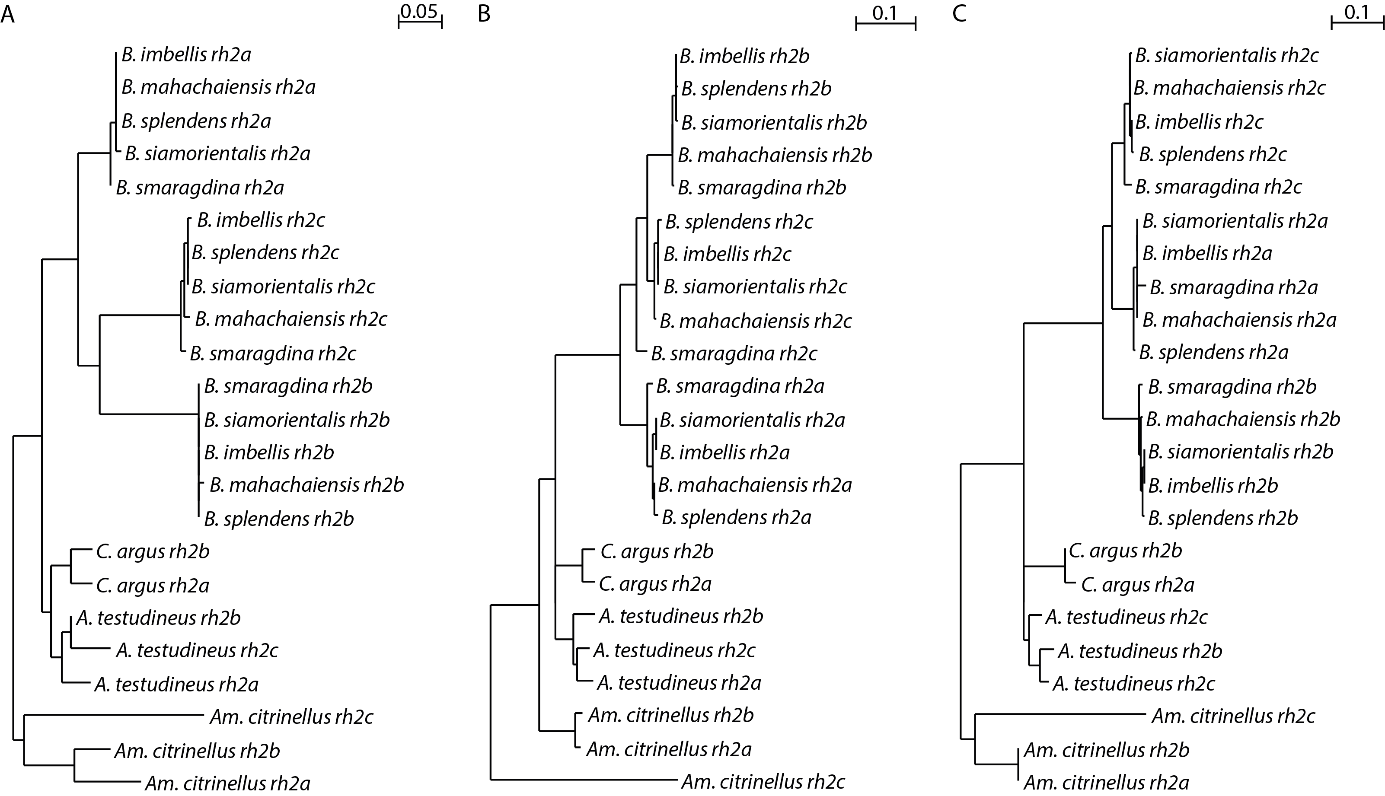


**Figure S3**: Phylogenetic reconstruction of base pairs 1-158 (A), 159-701 (B) and 702-1056 (C) of the coding sequences of *rh2* based on the recombination break points found using the Genetic Algorithm for Recombination Detection (GARD). Region


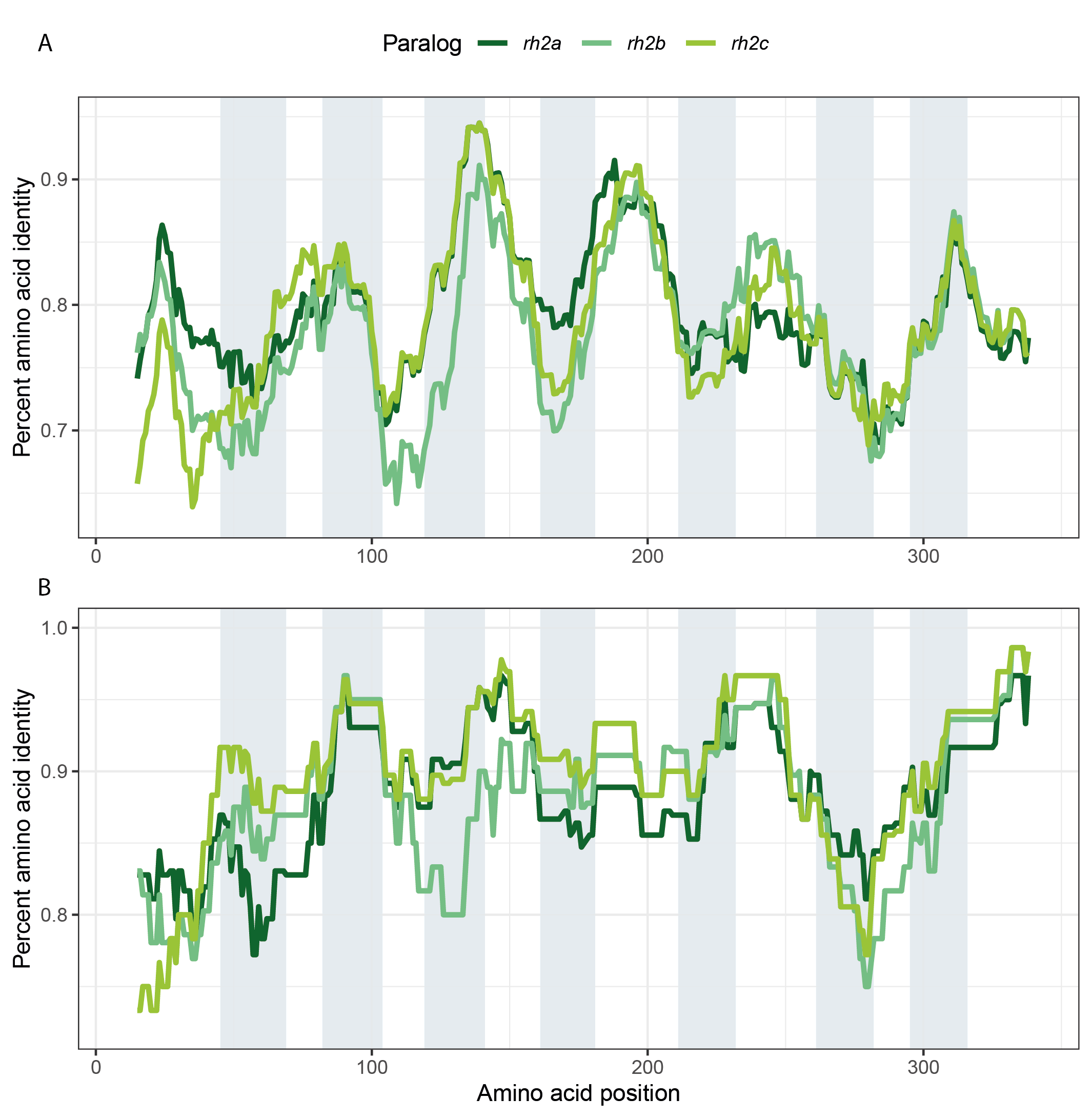


**Figure S4**: Sliding window analysis of *Betta* *rh2* amino acid sequence divergence. Each green line represents the mean divergence between one *rh2* paralog of all *Betta* species and *rh2* sequences of a set of representative teleost species (A) or the mean divergence between one *rh2* paralog of all *Betta* species and the remaining two paralogs in *Betta* (B). Grey areas indicate the position of transmembrane domains of *rh2*.


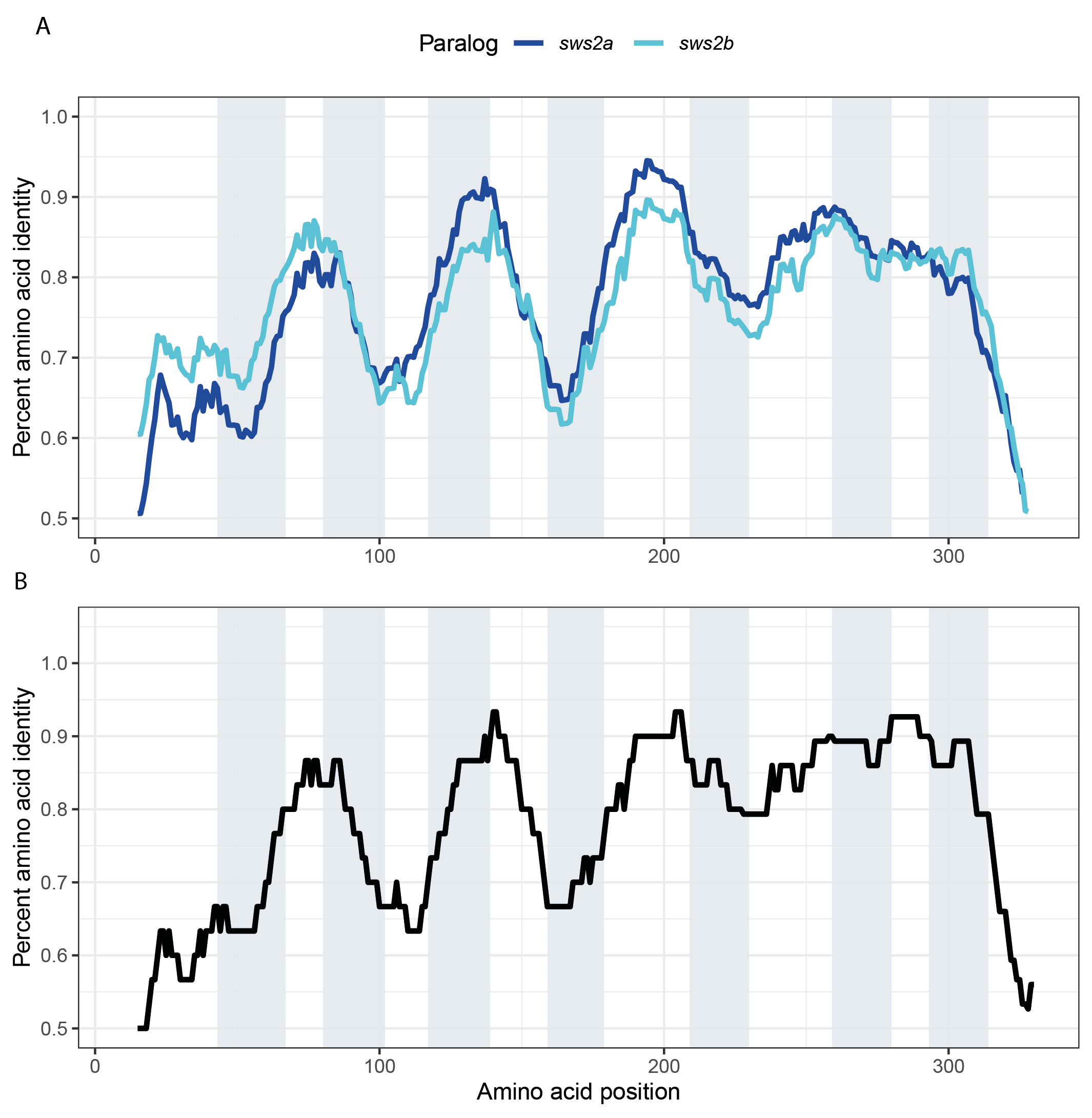


**Figure S5**: Sliding window analysis of *Betta* *sws2* amino acid sequence divergence. Each green blue represents the mean divergence between one *sws2* paralog of all *Betta* species and *sws2* sequences of a set of representative teleost species (A). The black line represents the mean divergence between the two *sws2* paralogs of all *Betta* species (B). Grey areas indicate the position of transmembrane domains of *rh2*.


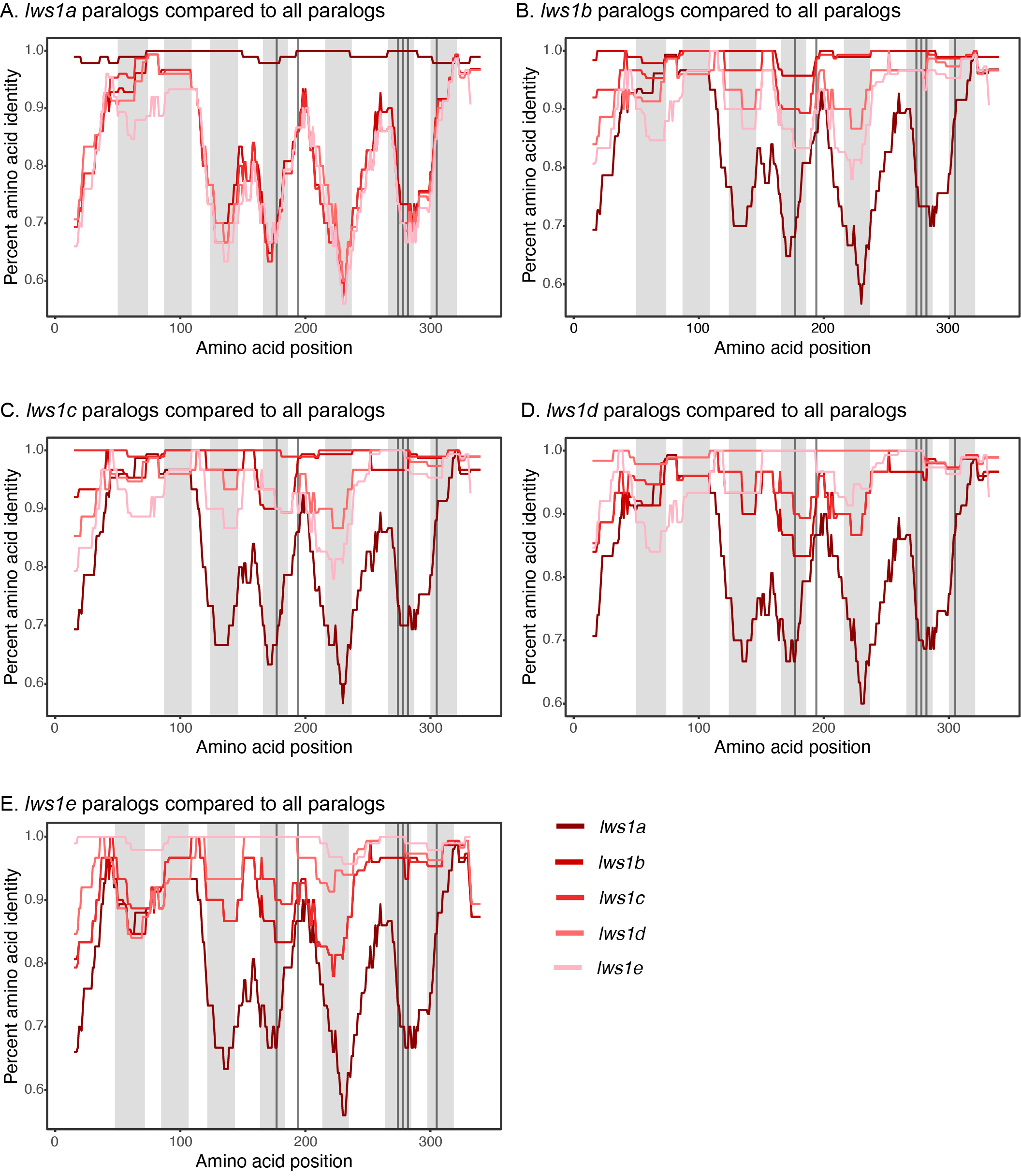


**Figure S6: Sliding window analysis of amino acid sequence divergence among the five *lws* paralogs in the genus *Betta* show that there observed pattern of divergence is not due to any particular paralog.** Each panel shows the mean divergence between one *lws* paralog of all *Betta* species and the remaining for paralogs in *Betta* (including against orthologs of the specific paralog). (A) *lws1a* (B) *lws1b*, (C) *lws1c*, (D) *lws1d*, and (E) *lws1e*. Grey areas indicate the position of the seven transmembrane domains of *lws*. Dark vertical bars indicate the positions of *lws* key tuning sites (based on Yokoyama et al. 2008).

**Table S1**: Estimated shift of maximal absorption between *rh2* genes in *Betta splendens* based on amino acid substitutions at sites of one known key tuning sites of *rh2* (Yokoyama et al. 2008).


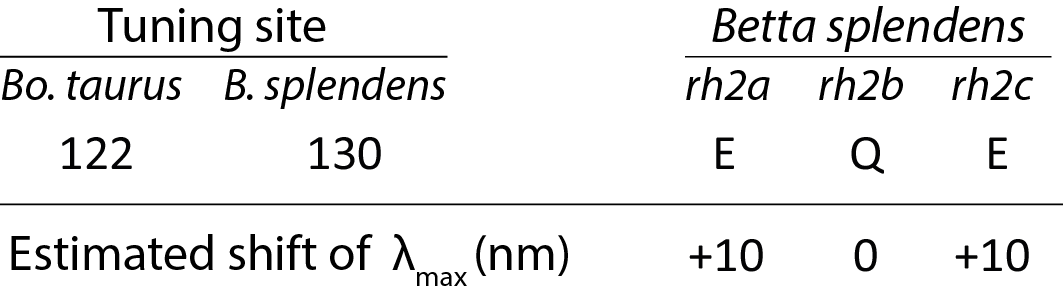


**Table S2**: Orthologs of *lws* opsin gene in outgroup teleost species used in the sliding window analysis of amino acid sequence divergence (see figure 3 in main text).

| **GenBank accession number** | ***Species*** | **Gene** |
| --- | --- | --- |
| DQ354578.1 | *Acanthopagrus butcheri* | long-wavelength sensitive opsin (LWS) |
| FJ711158.1 | *Anableps anableps* | LWS S180beta opsin |
| FJ711155.1 | *Anableps anableps* | LWS S180r opsin |
| AY660540.1 | *Astatotilapia burtoni* | putative red sensitive opsin, LWS |
| M90075.1 | *Astyanax fasciatus* | red-like visual pigment (red vision) |
| EU410457.1 | *Candidia barbatus* | red-sensitive pigment (LWS) |
| L11867.1 | *Carassius auratus* | red cone opsin |
| GQ168789.1 | *Carassius auratus* | long-wavelength sensitive opsin (LWS1) |
| AB055656.1 | *Cyprinus carpio* | red-sensitive opsin |
| NM_001002443.1 | *Danio rerio* | long-wave-sensitive (opn1lw2) |
| NM_131175.1 | *Danio rerio* | long-wave-sensitive (opn1lw1) |
| BT027981.1 | *Gasterosteus aculeatus* | Clone CNB93-C08 |
| AB158261.1 | *Girella punctata* | red opsin |
| AF316498.1 | *Hippoglossus hippoglossus* | red opsin |
| GQ221677.1 | *Jenynsia onca* | LWS S180 opsin |
| GQ221675.1 | *Jenynsia onca* | LWS S180 opsin |
| GQ221671.1 | *Jenynsia onca* | LWS S180r opsin |
| HM627007.1 | *Jordanella floridae* | LWS S180-1 opsin |
| HM627009.1 | *Jordanella floridae* | LWS S180-2 opsin |
| AY296740.1 | *Lucania goodei* | LWSA opsin |
| AY296741.1 | *Lucania goodei* | LWSB opsin |
| DQ088628.1 | *Melanochromis vermivorus* | putative red sensitive opsin, LWS |
| FJ940702.1 | *Melanotaenia australis* | long wave-sensitive opsin |
| AF247126.1 | *Metriaclima zebra* | putative red sensitive opsin, LWS |
| AY214150.1 | *Oncorhynchus gorbuscha* | LWS opsin |
| AY214140.1 | *Oncorhynchus keta* | LWS opsin |
| AY214145.1 | *Oncorhynchus kisutch* | LWS opsin |
| NM_001124320.1 | *Oncorhynchus mykiss* | LWS opsin |
| AY214155.1 | *Oncorhynchus nerka* | LWS opsin |
| AY214135.1 | *Oncorhynchus tshawytscha* | LWS opsin |
| AF247128.1 | *Oreochromis niloticus* | putative red sensitive opsin, LWS |
| AB223052.1 | *Oryzias latipes* | M/LWS type opsin, LLWS-B |
| AB223051.1 | *Oryzias latipes* | M/LWS type opsin, LLWS-A |
| AB107771.1 | *Plecoglossus altivelis* | red-sensitive opsin |
| AB098702.1 | *Plecoglossus altivelis* | putative red sensitive opsin, LWS |
| EU329433.1 | *Poecilia reticulata* | long wave-sensitive opsin S180 |
| DQ088627.1 | *Pseudotropheus sp. 'acei'* | putative red sensitive opsin, LWS |
| AB448425.1 | *Pundamilia pundamilia* | LWS, long wavelength-sensitive opsin |
| NM_001123705.1 | *Salmo salar* | long-wave-sensitive opsin |
| AF385826.1 | *Scophthalmus maximus* | red-sensitive opsin |
| GQ168786.1 | *Sinocyclocheilus anophthalmus* | long wavelength-sensitive cone opsin cl-1 |
| GQ168787.1 | *Sinocyclocheilus anophthalmus* | long wavelength-sensitive cone opsin cl-2 |
| GQ168768.1 | *Sinocyclocheilus jii* | long wavelength-sensitive cone opsin |
| AY598942.1 | *Takifugu rubripes* | red-sensitive pigment (LWS) |
| AY598943.1 | *Tetraodon nigroviridis* | red-sensitive pigment (LWS) |
| DQ088629.1 | *Tramitichromis intermedius* | putative red sensitive opsin, LWS |
| EU825688.1 | *Xiphophorus birchmanni* | Isolate LWS opsin |
| EU825686.1 | *Xiphophorus malinche* | Isolate LWS opsin |
| EU329480.1 | *Xiphophorus pygmaeus* | long wave-sensitive opsin |
| EU329478.1 | *Xiphophorus pygmaeus* | long wave-sensitive opsin S180 |
| EU410466.1 | *Zacco pachycephalus* | red-sensitive pigment (LWS) |

**Table S3**: Coding sequence evolution of the five *lws* paralogs in the genus *Betta* using codon-based models in PAML (Random Sites Model in codeml).

|  |  |  |  |  | Log-likelihood ratio tests | |
| --- | --- | --- | --- | --- | --- | --- |
| Gene | ns | ls | M0 tree length | ω_M0_ | M1a/M0 | M2a/M1a |
| lws1a | 5 | 358 | 0.044 | 0.223 | 0.00008^NS^ | <0.0001^NS^ |
| lws1b | 5 | 358 | 0.060 | 0.163 | 0.00002^NS^ | <0.0001^NS^ |
| lws1c | 5 | 358 | 0.050 | 0.000 | 0.00888^NS^ | 0.0009^NS^ |
| lws1d | 5 | 358 | 0.058 | 0.062 | 0.00005^NS^ | <0.0001^NS^ |
| lws1e | 5 | 357 | 0.055 | 0.022 | 0.00757^NS^ | <0.0001^NS^ |

NOTE.—ns, number of sequences; ls, length of sequences; NS, non-significant.

References

Kerpedjiev P, Abdennur N, Lekschas F, McCallum C, Dinkla K, Strobelt H, Luber JM, Ouellette SB, Azhir A, Kumar N (2018) HiGlass: web-based visual exploration and analysis of genome interaction maps. Genome biology 19:1

Yokoyama S, Yang H, Starmer WT (2008) Molecular basis of spectral tuning in the red- and green-sensitive (M/LWS) pigments in vertebrates. Genetics 179:2037
